# Long‐term safety and efficacy of ritlecitinib in adults and adolescents with alopecia areata and at least 25% scalp hair loss: Results from the ALLEGRO‐LT phase 3, open‐label study

**DOI:** 10.1111/jdv.20526

**Published:** 2025-01-23

**Authors:** C. Tziotzios, R. Sinclair, A. Lesiak, S. Mehlis, M. Kinoshita‐Ise, A. Tsianakas, X. Luo, E. H. Law, R. Ishowo‐Adejumo, R. Wolk, M. Sadrarhami, A. Lejeune

**Affiliations:** ^1^ St John's Institute of Dermatology King's College London London UK; ^2^ Sinclair Dermatology Melbourne Victoria Australia; ^3^ Department of Dermatology, Pediatric Dermatology and Oncology Medical University of Lodz Lodz Poland; ^4^ Laboratory of Autoinflammatory Genetic and Rare Skin Disorders, Department of Dermatology, Pediatric Dermatology and Oncology Medical University of Lodz Lodz Poland; ^5^ Division of Dermatology, Department of Medicine NorthShore University HealthSystem Evanston Illinois USA; ^6^ Department of Dermatology Kyorin University Faculty of Medicine Tokyo Japan; ^7^ Department of Dermatology Fachklinik Bad Bentheim Bad Bentheim Germany; ^8^ Pfizer Inc Groton Connecticut USA; ^9^ Pfizer Inc New York City New York USA; ^10^ Pfizer Inc Collegeville Pennsylvania USA; ^11^ Pfizer Inc Paris France

## Abstract

**Background:**

ALLEGRO‐LT is an ongoing, long‐term, open‐label, multicentre, phase 3 study of ritlecitinib in adults and adolescents with alopecia areata (AA).

**Objectives:**

To evaluate ritlecitinib safety and efficacy through Month 24 in patients with AA and ≥25% scalp hair loss.

**Methods:**

ALLEGRO‐LT enrolled rollover patients who previously received study intervention in either ALLEGRO phase 2a or 2b/3 studies and de novo patients who had not received treatment in either study. The de novo cohort results are reported here. Patients aged ≥12 years with AA and ≥25% scalp hair loss received a daily, 4‐week 200‐mg ritlecitinib loading dose, followed by daily 50‐mg ritlecitinib. Analyses are based on data up to the cut‐off (December 2022). Efficacy outcomes included proportions of patients achieving Severity of Alopecia Tool (SALT) scores ≤20 and ≤10, Patient Global Impression of Change (PGI‐C) score of ‘moderately improved’ or ‘greatly improved’ and eyebrow assessment (EBA) and eyelash assessment (ELA) response (≥2‐grade improvement from baseline or normal score in patients with abnormal baseline EBA/ELA).

**Results:**

Mean (SD) ritlecitinib exposure among the 449 de novo patients enrolled was 728.7 (273.81) days. At Month 24 (as observed), 73.5% and 66.4% of patients achieved SALT score ≤20 and ≤10; 82.4% had PGI‐C response; 60.8% and 65.7% had EBA and ELA response. 86.1% of patients reported treatment‐emergent adverse events (AEs); most were mild or moderate in severity, with the most frequent being positive SARS‐CoV‐2 test (24.2%), headache (20.8%) and pyrexia (13.0%). Rates of serious AEs, severe AEs and treatment discontinuations were 4.9%, 6.0% and 6.5%, respectively. Herpes zoster infection occurred in six patients, serious infections in four, malignancies (excluding nonmelanoma skin cancer) in three and major adverse cardiovascular events in three.

**Conclusions:**

In patients with AA and ≥25% scalp hair loss, ritlecitinib demonstrated clinical efficacy and had an acceptable safety profile with long‐term treatment.

**Clinical Trial Registration:**

ClinicalTrials.gov NCT04006457.

## Graphical abstract

ALLEGRO‐LT (NCT04006457) is an open‐label, phase 3 study investigating the long‐term safety and efficacy of ritlecitinib in patients with alopecia areata (AA). In this analysis in 449 de novo patients with AA and ≥25% scalp hair loss, ritlecitinib demonstrated clinical efficacy and had an acceptable safety profile through Month 24.

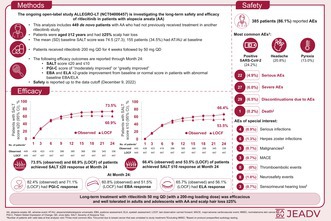

**Long‐term safety and efficacy of ritlecitinib in adults and adolescents with alopecia areata and at least 25% scalp hair loss: Results from the ALLEGRO‐LT phase 3, open‐label study** by Tziotzios et al.

Video

**Why was the study undertaken?**

- Alopecia areata (AA) can be chronic, and long‐term therapy is needed for optimal disease control in some patients, but effective systemic treatment options are limited.
- Efficacy data on new systemic treatments for AA in patients with 25% or more scalp hair loss are currently very limited.

**What does this study add?**

- Long‐term treatment up to 24 months with ritlecitinib 50 mg daily (with a 200‐mg loading dose for 4 weeks daily) was efficacious in all subgroups of patients stratified by baseline scalp hair loss.
- The long‐term safety profile of ritlecitinib in this study was consistent with that previously reported.

**What are the implications of this study for disease understanding and/or clinical care?**

- Ritlecitinib demonstrated clinical efficacy with long‐term treatment, including in patients with extensive hair loss at baseline, and the long‐term safety profile of ritlecitinib in this study was consistent with previously published integrated data.
- Higher response rates in patients with less extensive scalp hair loss indicate that therapeutic intervention early in the disease course may lead to improved chance of treatment success than waiting for extensive scalp hair loss to occur.

## INTRODUCTION

Alopecia areata (AA) is an autoimmune disease with an underlying immuno‐inflammatory pathogenesis characterized by non‐scarring hair loss ranging from small patches of hair loss to complete loss of scalp, face and/or body hair.^1^ The global prevalence of AA has been estimated at approximately 2%,^2^ and patients with AA may experience psychological and psychosocial symptoms that negatively impact their quality of life.^3^, ^4^, ^5^, ^6^

Extensive AA subtypes include alopecia totalis (AT; complete loss of scalp hair) and alopecia universalis (AU; complete loss of scalp, face and body hair).^2^ Patients with AT and AU are often refractory to treatment with off‐label therapies,^7^, ^8^ and patients with more extensive hair loss at baseline may be less likely to have a robust response to treatment.^9^, ^10^, ^11^, ^12^

The pathogenesis of AA involves the loss of immune privilege at the hair follicle (HF) and recognition of exposed HF autoantigens by T‐cell receptors (TCRs) on autoreactive CD8+ T cells.^13^, ^14^, ^15^ Interferon‐γ and interleukin‐15, which transduce signals through the Janus kinase–signal transducer and activator of transcription (JAK–STAT) pathway, are involved in the activation and proliferation of autoreactive T cells.^16^, ^17^, ^18^ Downstream signalling by exposed HF autoantigens via TCRs involves the tyrosine kinase expressed in hepatocellular carcinoma (TEC) family of kinases, which have also been implicated in the pathogenesis of AA.^18^, ^19^, ^20^, ^21^

AA may be chronic, with long‐term therapy needed for optimal disease control in some patients, yet effective systemic treatment options are limited. Currently, there are three approved oral treatments for severe AA: baricitinib and deuruxolitinib, which are JAK1/2 inhibitors,^22^, ^23^ and ritlecitinib, a selective dual inhibitor of JAK3 and the TEC family of kinases.^24^ All treatments are approved for adult patients with severe AA, while ritlecitinib is the only treatment approved for adolescent patients (aged ≥12 years).

Few definitions of AA severity have been proposed for use in clinical practice.^25^ In clinical trials, the Severity of Alopecia Tool (SALT) is commonly used as a measure of AA severity based on the extent of scalp hair loss.^26^, ^27^ This scale does not consider hair loss in other areas, such as eyebrows or eyelashes, or the psychosocial impact on patients.^3^, ^4^, ^5^, ^6^, ^25^, ^28^, ^29^, ^30^, ^31^ The AA Scale is a disease severity scale for use in clinical practice that includes eyebrow and eyelash involvement, inadequate response after at least 6 months of treatment, a positive hair pull test and impact on psychosocial functioning, in addition to scalp hair loss.^32^ Using the AA Scale, patients with 50% to 100% scalp hair loss are classified as having severe AA; those with 21% to 49% scalp hair loss are classified as having moderate AA, but this may be increased to severe if ≥1 of the additional features are present.^32^ Despite this, efficacy data on new systemic treatments for AA in patients with less than 50% scalp hair loss are currently limited.

The pivotal ALLEGRO phase 2b/3 study (ALLEGRO‐2b/3; NCT03732807) demonstrated that ritlecitinib was efficacious and well tolerated in patients aged ≥12 years with AA and ≥50% scalp hair loss, as measured by SALT.^24^ In ALLEGRO‐2b/3, 23% of patients who received ritlecitinib 50 mg daily (QD), compared with 2% in the placebo group, achieved the primary endpoint of SALT ≤20 at Week 24, increasing to 43% at Week 48.^24^

The ongoing open‐label study ALLEGRO‐LT (NCT04006457) is investigating the long‐term safety and efficacy of ritlecitinib in patients with AA. ALLEGRO‐LT includes patients rolled over from previous index studies of ritlecitinib and a de novo cohort of patients aged ≥12 years with no previous ritlecitinib treatment and have ≥25% scalp hair loss, as measured by SALT, representing a broader patient population than prior AA phase 3 studies.^22^, ^24^

Here we report a long‐term safety and efficacy analysis of de novo patients in ALLEGRO‐LT, including the effect of baseline SALT score on efficacy outcomes.

## MATERIALS AND METHODS

### Study design

ALLEGRO‐LT is an ongoing, phase 3, open‐label, multicentre study investigating the long‐term safety and efficacy of ritlecitinib in adult and adolescent patients with AA, enrolled into two arms: (1) rollover patients who had received treatment in the ALLEGRO phase 2a proof‐of‐concept study (NCT04417864) or ALLEGRO‐2b/3 and (2) de novo patients who had not received ritlecitinib treatment in either study. Rollover patients received ritlecitinib 50 mg QD. De novo patients received ritlecitinib 200 mg QD for 4 weeks followed by 50 mg QD. The 4‐week, 200‐mg loading‐dose regimen demonstrated an acceptable safety profile in the ALLEGRO phase 2a study^33^; its use was based on the hypothesis that maximal inhibition of the relevant immunomodulatory pathways at initiation of treatment can accelerate clinical response, which can then be maintained by the subsequent, lower maintenance dose.

The current analysis includes patients in the de novo cohort only, and the data cut‐off was 9 December 2022. Interim results are subject to change as additional data are collected and analysed in the ongoing study.

### Patients

The de novo cohort included patients aged ≥12 years with a diagnosis of AA. All patients in this analysis had ≥25% scalp hair loss due to AA (including AT and AU), as measured by SALT, and no evidence of terminal hair regrowth within 6 months at both the screening and baseline visits. The duration of the current episode of hair loss was required to be ≤10 years. Adolescent patients (aged 12–17 years) were required to have a SALT score≤20 at Month 6 to continue in the study.

Patients with any previous use of a JAK inhibitor were excluded. Detailed inclusion and exclusion criteria are outlined in Table S1.

Patients were required to discontinue any treatments that could affect AA; prohibited concomitant medications and treatments are outlined in Table S2. Unless a prohibited medication or treatment, patients were permitted to be administered any other medications necessary for the treatment of concomitant medical disorders as deemed necessary by the treating physician.

### Outcomes

The primary objective of the study was to assess the long‐term safety of ritlecitinib in adult and adolescent patients with AA. Safety endpoints reported here include the incidence of treatment‐emergent adverse events (AEs), serious AEs and AEs leading to discontinuation through the data cut‐off date. Adjudication committees (external experts, blinded to treatment and independent from the study sponsor) were established to obtain objective assessment of opportunistic infections, malignancies, cardiovascular and thromboembolic events, and neurological and audiological events.

The secondary objective was to assess the long‐term efficacy of ritlecitinib. Secondary endpoints reported here included response at Month 24 based on SALT score≤20 (≤20% scalp hair loss), SALT score≤10 (≤10% scalp hair loss), Patient Global Impression of Change (PGI‐C) score of ‘moderately improved’ or ‘greatly improved’ since baseline, ≥2‐grade improvement from baseline or a score of 3 in eyebrow assessment (EBA) in patients with abnormal EBA score (defined as <3) at baseline and ≥2‐grade improvement from baseline or a score of 3 in eyelash assessment (ELA) in patients with abnormal ELA score (defined as <3) at baseline.

### Statistical analysis

Safety analyses were performed on the safety population (all patients who received ≥1 dose of study intervention) to the time of data cut‐off and are reported descriptively.

Efficacy data are presented as observed and imputed using last observation carried forward (LOCF) to account for missing data. LOCF was applied to each visit for all patients with missing data, except for those who had not yet reached that analysis visit. These interim, pooled analyses are descriptive in nature and there was no formal hypothesis testing. The 95% confidence intervals (CIs) were calculated based on normal approximation. Patients were stratified by extent of hair loss at baseline, as measured by SALT score, into four strata: SALT 25 to <50 (25% to <50% scalp hair loss), SALT 50 to <75 (50% to <75% scalp hair loss), SALT 75 to <100 (75% to <100% scalp hair loss) and SALT 100 (100% scalp hair loss).

## RESULTS

### Patients disposition and demographics

The de novo cohort in ALLEGRO‐LT comprised 449 patients who received ritlecitinib 200‐mg QD loading dose for 4 weeks followed by 50 mg QD. At baseline, there were 119, 76, 99 and 155 patients in the SALT score categories of 25 to <50, 50 to <75, 75 to <100 and 100, respectively. A total of 124 patients (27.6%) patients discontinued the study prior to end of study, with the most common reasons being withdrawal by participant (*n* = 30 [6.7%]), lack of efficacy (*n* = 21 [4.7%]) and an AE (*n* = 19 [4.2%]). Eighteen adolescent patients (4.0%) discontinued due to not meeting the protocol continuation criteria (i.e. not achieving a SALT score≤20 at Month 6). Patient disposition by baseline SALT categories is described in Table 1.

**TABLE 1:** Patient disposition of the de novo cohort by baseline SALT categories.

	Baseline SALT score 25 to <50 (*n* = 119)	Baseline SALT score 50 to <75 (*n* = 76)	Baseline SALT score 75 to <100 (*n* = 99)	Baseline SALT score 100 (*n* = 155)	Total (*N* = 449)
Assigned to treatment	119	76	99	155	449
Treated	119 (100.0)	75 (98.7)	98 (99.0)	155 (100.0)	447 (99.6)
Not treated	0	1 (1.3)	1 (1.0)	0	2 (0.4)
Discontinued	30 (25.2)	16 (21.1)	18 (18.2)	60 (38.7)	124 (27.6)
Adverse event	4 (3.4)	5 (6.6)	1 (1.0)	9 (5.8)	19 (4.2)
Death	1 (0.8)	0	0	0	1 (0.2)
Lack of efficacy	4 (3.4)	0	2 (2.0)	15 (9.7)	21 (4.7)
Lost to follow‐up	6 (5.0)	2 (2.6)	2 (2.0)	6 (3.9)	16 (3.6)
Noncompliance with study drug	1 (0.8)	0	0	3 (1.9)	4 (0.9)
Physician decision	1 (0.8)	0	0	1 (0.6)	2 (0.4)
Pregnancy	2 (1.7)	3 (3.9)	0	2 (1.3)	7 (1.6)
Withdrawal by participant	9 (7.6)	2 (2.6)	7 (7.1)	12 (7.7)	30 (6.7)
No longer meets eligibility criteria^a^	1 (0.8)	3 (3.9)	5 (5.1)	9 (5.8)	18 (4.0)
Other	1 (0.8)	1 (1.3)	1 (1.0)	3 (1.9)	6 (1.3)
Analysed for efficacy	119 (100.0)	76 (100.0)	99 (100.0)	155 (100.0)	449 (100.0)
Analysed for safety	119 (100.0)	75 (98.7)	98 (99.0)	155 (100.0)	447 (99.6)

*Note*: All data are *n* or *n* (%).

Abbreviation: SALT, Severity of Alopecia Tool.

^a^ Patients who did not meet the protocol continuation criteria for adolescents (i.e. did not achieve a SALT ≤20 score at Month 6).

Baseline demographics and clinical characteristics are shown in Table 2. The mean age (standard deviation [SD]) was 32.9 (14.0) years, and 76 patients (16.9%) were adolescents (aged 12–17 years). The mean (SD) baseline SALT score was 74.5 (27.3); 155 patients (34.5%) had AT and/or AU, defined as a baseline SALT score of 100. The median duration of AA since diagnosis and duration of current episode were 6.1 and 1.9 years, respectively. Overall, 73.5% and 65.9% of patients, respectively, had abnormal EBA and ELA scores at baseline.

**TABLE 2:** Demographic and baseline characteristics of the overall de novo cohort and by individual baseline SALT categories.

	Baseline SALT score 25 to <50 (*n* = 119)	Baseline SALT score 50 to <75 (*n* = 76)	Baseline SALT score 75 to <100 (*n* = 99)	Baseline SALT score 100 (*n* = 155)	Total (*N* = 449)
Age, mean (SD), years	34.4 (14.6)	31.6 (14.3)	31.3 (13.6)	33.3 (13.5)	32.9 (14.0)
Age group, *n* (%)					
12–17 years	17 (14.3)	17 (22.4)	24 (24.2)	18 (11.6)	76 (16.9)
≥18 years	102 (85.7)	59 (77.6)	75 (75.8)	137 (88.4)	373 (83.1)
Female, *n* (%)	77 (64.7)	48 (63.2)	65 (65.7)	99 (63.9)	289 (64.4)
Ethnic background, *n* (%)					
White	81 (68.1)	56 (73.7)	55 (55.6)	118 (76.1)	310 (69.0)
Black or African American	2 (1.7)	3 (3.9)	7 (7.1)	4 (2.6)	16 (3.6)
Asian	24 (20.2)	12 (15.8)	33 (33.3)	27 (17.4)	96 (21.4)
American Indian or Alaska Native	1 (0.8)	0	2 (2.0)	2 (1.3)	5 (1.1)
Native Hawaiian or other Pacific Islander	2 (1.7)	0	0	0	2 (0.4)
Multiracial	2 (1.7)	2 (2.6)	0	3 (1.9)	7 (1.6)
Not reported	7 (5.9)	3 (3.9)	2 (2.0)	1 (0.6)	13 (2.9)
Duration of AA since diagnosis, mean (SD), years	8.8 (9.8)	9.2 (9.3)	10.2 (10.8)	10.7 (10.6)	9.8 (10.2)
Duration of current AA episode, mean (SD), years	2.5 (2.4)	2.8 (2.4)	2.4 (2.2)	3.6 (2.9)	2.9 (2.6)
Type of current episode, *n* (%)^a^					
AT/AU	0	0	0	155 (100.0)	155 (34.5)
AT	0	0	0	75 (48.4)	75 (16.7)
AU	0	0	0	73 (47.1)	73 (16.3)
Non‐AT/AU	119 (100.0)	76 (100.0)	99 (100.0)	0	294 (65.5)
Baseline SALT score, mean (SD)	36.2 (7.6)	61.1 (7.0)	91.1 (7.8)	100.0 (0.0)	74.5 (27.3)
Without normal EBA, *n* (%)	58 (48.7)	42 (55.3)	79 (79.8)	151 (97.4)	330 (73.5)
Without normal ELA, *n* (%)	49 (41.2)	30 (39.5)	73 (73.7)	144 (92.9)	296 (65.9)

Abbreviations: AA, alopecia areata; AT, alopecia totalis; AU, alopecia universalis; SALT, Severity of Alopecia Tool; SD, standard deviation.

^a^ Patients in the AT/AU group had a SALT score of 100 at baseline regardless of the category in the AA history case report form.

Patients with higher SALT scores at baseline tended to have longer duration of AA since diagnosis and duration of current episode. The proportions of patients with eyebrow or eyelash involvement tended to be higher in patients with greater extent of scalp hair loss at baseline. However, in the SALT 25 to <50 category, substantial proportions of patients (48.7% for EBA and 41.2% for ELA) had abnormal EBA/ELA scores at baseline.

### Treatment exposure

The mean (SD) duration of treatment was 728.7 (273.81) days, and most patients (74.5%) had ≥24 months of treatment.

### Efficacy

#### SALT ≤20 and <10 response at Month 24

**FIGURE 1:**
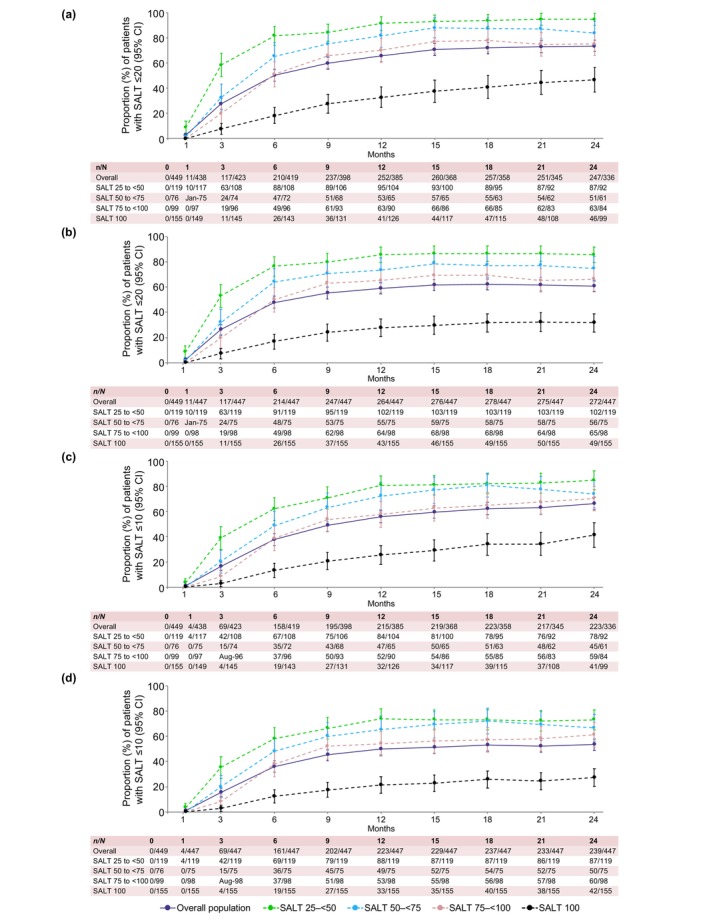
SALT ≤20 and ≤10 response rates to Month 24 for overall population and individual baseline SALT categories. SALT ≤20 response as observed (a) and LOCF (b); SALT ≤10 response as observed (c) and LOCF (d). LOCF, last observation carried forward; SALT, Severity of Alopecia Tool. [Correction added on 22 April 2025, after first online publication: The figure has been updated with the x‐axis label (Months).]

The proportion of SALT ≤20 and ≤10 responders generally increased over time. In patients in the SALT 100 category, the proportion of SALT ≤20 responders was the lowest of all the SALT categories through Month 24 using observed data and when analysing data using LOCF (Figure 1).

Out of 336 patients with observed data at Month 24, 247 (73.5%) had a SALT score≤20 (Figure 1a). SALT ≤20 response at Month 24 in the baseline categories SALT 25 to <50, 50 to <75, 75 to <100 and 100 was 94.6%, 83.6%, 75.0% and 46.5%, respectively. Out of 447 patients included in the LOCF analysis, 272 (60.9%) had a SALT score≤20 at Month 24. LOCF data for the individual SALT categories are shown in Figure 1b.

Out of 336 patients with observed data at Month 24, 223 (66.4%) had a SALT score≤10 (Figure 1c). SALT ≤10 responses at Month 24 in the baseline categories SALT 25 to <50, 50 to <75, 75 to <100 and 100 were 84.8%, 73.8%, 70.2% and 41.4%, respectively.

Out of 447 patients included in the LOCF analysis, 239 patients (53.5%) patients had a SALT score≤10 at Month 24. LOCF data for the individual SALT categories are shown in Figure 1d.

#### PGI‐C response at Month 24

**FIGURE 2:**
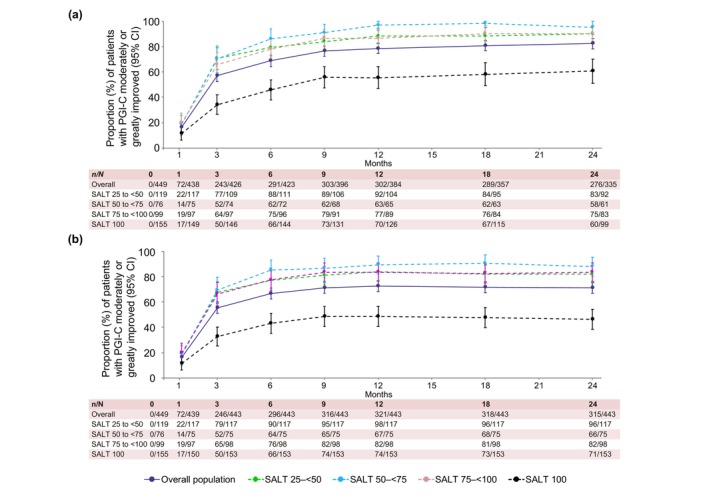
PGI‐C response rates defined as scores of ‘moderately improved’ or ‘greatly improved’ to Month 24 for overall population and individual baseline SALT categories for observed (a) and LOCF (b) data. LOCF, last observation carried forward; PGI‐C, Patient Global Impression of Change; SALT, Severity of Alopecia Tool. [Correction added on 22 April 2025, after first online publication: The figure has been updated with the x‐axis label (Months).]

Out of 335 patients with observed data at Month 24, 276 (82.4%) had a PGI‐C score of ‘moderately improved’ or ‘greatly improved’ from baseline (Figure 2a). PGI‐C responses at Month 24 in the baseline SALT categories of 25 to <50, 50 to <75, 75 to <100 and 100 were 90.2%, 95.1%, 90.4% and 60.6%, respectively. LOCF data for PGI‐C response are shown in Figure 2b.

#### EBA and ELA response at Month 24

**FIGURE 3:**
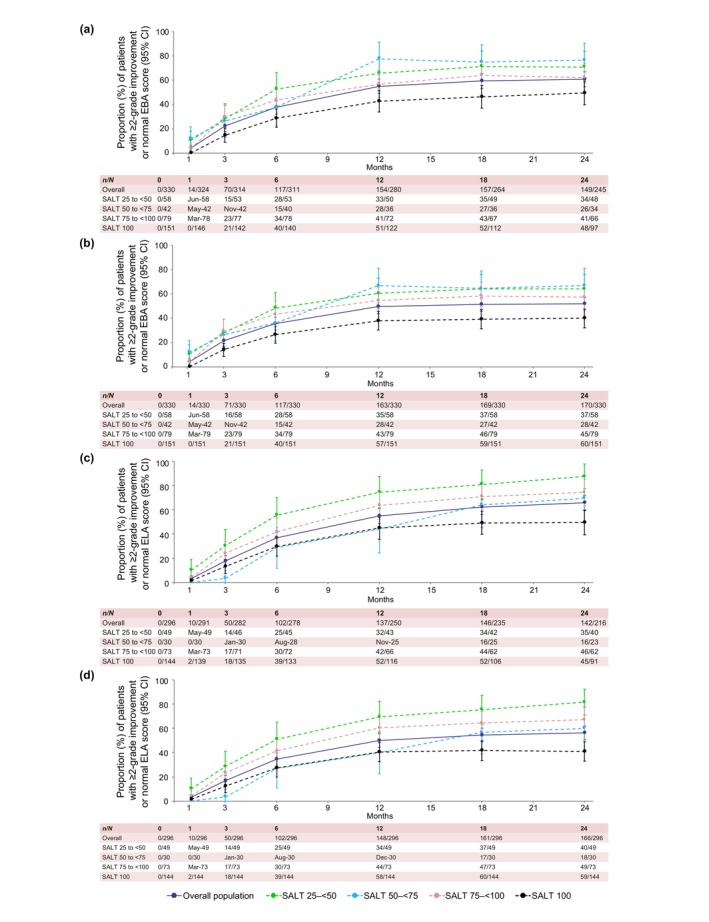
EBA and ELA response rates to Month 24 for overall population and individual baseline SALT categories. Patients with abnormal EBA score at baseline with ≥2‐grade improvement from baseline or a score of 3 in EBA as observed (a) and LOCF (b); patients with abnormal ELA score at baseline with ≥2‐grade improvement from baseline or a score of 3 in ELA as observed (c) and LOCF (d). EBA, eyebrow assessment; ELA, eyelash assessment; LOCF, last observation carried forward; SALT, Severity of Alopecia Tool. [Correction added on 22 April 2025, after first online publication: The figure has been updated with the x‐axis label (Months).]

Out of 245 patients who had abnormal EBA scores at baseline and had observed data at Month 24, 149 (60.8%) had a ≥2‐grade improvement from baseline or a normal EBA score (Figure 3a). EBA responses at Month 24 in the baseline categories SALT 25 to <50, 50 to <75, 75 to <100 and 100 were 70.8%, 76.5%, 62.1% and 49.5%, respectively.

Out of 216 patients who had abnormal ELA scores at baseline and had observed data at Month 24, 142 (65.7%) had a ≥2‐grade improvement from baseline or a normal ELA score (Figure 3c). ELA responses at Month 24 in the baseline categories SALT 25 to <50, 50 to <75, 75 to <100 and 100 were 87.5%, 69.6%, 74.2% and 49.5%, respectively.

LOCF data for EBA and ELA response are shown in Figure 3b,d, respectively.

### Safety and tolerability

Among 447 patients included in the safety analysis, 385 (86.1%) experienced ≥1 AE. Most AEs were mild or moderate in severity (94%) (Table 3). The most common AEs were a positive SARS‐CoV‐2 test (24.2%), headache (20.8%) and pyrexia (13.0%).

**TABLE 3:** Overall safety summary and most frequent treatment‐emergent AEs.

	Total (*n* = 447)
Patients evaluable for AEs	447
No. of AEs	2019
Patients with AEs, *n* (%)	385 (86.1)
Patients with SAEs, *n* (%)	22 (4.9)
Patients with severe AEs, *n* (%)	27 (6.0)
Deaths^a^	1
Patients discontinued from study or study drug due to AEs, *n* (%)	29 (6.5)
Patients with temporary discontinuation due to AEs, *n* (%)	122 (27.3)
Most frequent AEs, *n* (%)^b^	
SARS‐CoV‐2 test positive	108 (24.2)
Headache	93 (20.8)
Pyrexia	58 (13.0)
Acne	54 (12.1)
Nasopharyngitis	52 (11.6)
Fatigue	46 (10.3)
Cough	43 (9.6)
Upper respiratory tract infection	41 (9.2)
Urticaria	37 (8.3)
Oropharyngeal pain	36 (8.1)
Urinary tract infection	27 (6.0)
Nausea	25 (5.6)
Diarrhoea	24 (5.4)
COVID‐19	23 (5.1)
Nasal congestion	23 (5.1)

Abbreviations: AE, adverse event; SAE, serious adverse event.

^a^ One death occurred due to breast cancer that was deemed by the investigator not to be related to the study drug.

^b^ Occurring in ≥5% of patients.

Temporary discontinuation from treatment due to AEs occurred in 122 patients (27.3%), and 29 (6.5%) permanently discontinued the study due to AEs, with the two most common being pregnancy (occurring in seven patients [1.6%]) and latent tuberculosis (occurring in three patients [0.7%]).

Twenty‐two patients (4.9%) experienced a serious AE, and one death (0.2%) occurred in the study due to breast cancer. There were four (0.9%) serious infections and no opportunistic infections. Herpes zoster was reported in six patients (1.3%) and herpes simplex in 12 (2.7%) (Table 4). There were two (0.4%) cases of nonmelanoma skin cancer and three (0.7%) other malignancies (one each of breast cancer, testicular cancer and thyroid cancer). There were three (0.7%) cases of MACE (including 2 [0.4%] cases of acute myocardial infarction and one [0.2%] case of retinal artery occlusion) and no thromboembolic events reported. One (0.2%) case of peripheral neuropathy, seven (1.6%) cases of paresthesia and dysesthesia and three (0.7%) cases of sensorineural hearing loss were reported.

**TABLE 4:** AEs of special interest.

n (%)	Total (*n* = 447)
Serious infections	4 (0.9)
COVID‐19	1 (0.2)
COVID‐19 pneumonia	1 (0.2)
Latent tuberculosis	1 (0.2)
Septic shock	1 (0.2)
Vulval abscess	1 (0.2)
Opportunistic infections	0
Herpes zoster	6 (1.3)
Herpes simplex	12 (2.7)
Malignancies, excluding NMSC	3 (0.7)
Breast cancer	1 (0.2)
Papillary thyroid cancer	1 (0.2)
Testicular cancer	1 (0.2)
NMSC	2 (0.4)
All MACE	3 (0.7)
Acute myocardial infarction	2 (0.4)
Retinal artery occlusion	1 (0.2)
Thromboembolic events	0
Peripheral neuropathy	1 (0.2)
Paresthesia and dysesthesia	7 (1.6)
Sensorineural hearing loss	3 (0.7)

Abbreviations: AE, adverse event; MACE, major adverse cardiovascular event; NMSC, nonmelanoma skin cancer.

## DISCUSSION

We report a long‐term safety and efficacy analysis of ritlecitinib in the de novo cohort from the open‐label ALLEGRO‐LT study, which included participants with ≥25% scalp hair loss. At Month 24, 73.5% and 66.4% of patients with observed data, and 60.9% and 53.5% of patients in the LOCF analysis, had SALT scores of ≤20 and ≤10, respectively, demonstrating the efficacy of ritlecitinib over a 24‐month period.

Improvements in SALT response were seen in all baseline SALT categories, including in patients with AT and AU. The highest response rates were seen in patients with scalp hair loss between 25% and 50%, including for SALT ≤10. However, it should be noted that patients with SALT 25 to <50 at baseline started the study with SALT scores that were closer to the SALT ≤20 and SALT ≤10 thresholds, conceivably making these endpoints easier to achieve. Patients with SALT 25 to <50 at baseline also had a shorter duration of AA since diagnosis at baseline. Overall, the higher response rates in patients with less extensive scalp hair loss indicate that therapeutic intervention early in the disease course may lead to a higher chance of treatment response than waiting for extensive scalp hair loss to occur.^11^, ^34^, ^35^

This study included patients with at least 25% hair loss at baseline, in contrast to the ALLEGRO‐2b/3 study, which included patients with at least 50% scalp hair loss at baseline; that is, mean baseline SALT score was slightly lower in this study versus ALLEGRO‐2b/3 (74.5 vs. 88.3–93.0). In ALLEGRO‐2b/3, 40% and 33% of patients who received 50 mg of ritlecitinib QD with a 200‐mg QD loading dose had a SALT score ≤20 and ≤10, respectively, at Week 48.^24^ Treatment responses at Week 48 were similar with and without a loading dose, suggesting that the loading dose used in the ALLEGRO‐LT de novo cohort would not impact the efficacy of longer‐term ritlecitinib treatment. Results from ALLEGRO‐LT support the efficacy of ritlecitinib in the broader population of patients with AA and demonstrate that treatment response continues to increase with long‐term treatment beyond 48 weeks, particularly in patients with AT or AU. Specifically, response rates in patients with a baseline SALT score of 100 continued to increase, with 46% and 41% SALT ≤20 and ≤10 response rates, respectively, at Month 24. These results suggest that ritlecitinib is efficacious in patients with extensive hair loss at baseline, although these patients may require longer treatment to achieve additional hair regrowth. In a separate analysis, over 90% of patients in ALLEGRO‐LT were shown to sustain their clinical response to ritlecitinib through Month 24, including patients with AT or AU.^36^

Regrowth of eyebrows and eyelashes was observed in patients across all baseline SALT categories, with over 60% of patients with eyebrow or eyelash involvement at baseline achieving an EBA or ELA response at Month 24 (as observed). Eyebrow and eyelash hair loss can have negative physical consequences, including eye irritation, along with psychosocial symptoms, which can have a profound detrimental impact on quality of life for patients with AA.^5^, ^28^, ^29^, ^30^, ^31^ In the SALT 25 to <50 category, 48.7% and 41.2% of patients had abnormal baseline EBA and ELA scores, respectively. These patients would be classified as having severe disease using the AA Scale, despite having <50% scalp hair loss.^32^ In this subset of patients, 70.8% and 87.5% achieved an EBA and ELA response, respectively, at Month 24.

High levels of patient‐reported improvement in AA, as measured by PGI‐C at Month 24, were seen across all baseline SALT categories, including in patients with AT or AU. There were no appreciable differences in PGI‐C scores between the SALT categories, except for SALT 100 (>90% for all SALT <100 categories vs. 60.6% for SALT 100), indicating similar levels of treatment satisfaction, regardless of baseline scalp hair loss. Patients' perception of AA improvement and satisfaction with treatment may be affected by factors including extent and duration of hair loss, level of acceptance of and coping strategies for hair loss, experience with previous treatments and treatment expectations. Expectations for hair regrowth following treatment have been shown to vary based on patients' existing hair loss,^37^ and the relationship between AA severity and the extent of health‐related quality of life impairment has been observed to vary across studies.^6^, ^38^

A full overview of the safety of ritlecitinib has been previously published. The long‐term safety profile of ritlecitinib in this study was consistent with that previously reported.^24^, ^39^ AEs were generally mild or moderate and did not require treatment interruption or permanent discontinuation. The global COVID‐19 outbreak occurred during the trial, which resulted in a positive SARS‐CoV‐2 test being the most frequent AE observed during the study. Overall, rates of serious infections, herpes zoster and herpes simplex were low, and there were no cases of opportunistic infections. Rates of malignancies and MACE were also low.

This analysis has some limitations. The study was not designed to identify differences in efficacy between baseline SALT categories, and the numbers of patients in the individual SALT category subgroups were relatively small. Also, ALLEGRO‐LT is an open‐label study with no blinding or placebo arm.

In summary, long‐term treatment with ritlecitinib 50 mg QD (with a 200‐mg loading dose) was efficacious and well tolerated in adults and adolescents with AA and scalp hair loss ≥25%. Efficacy was observed in all subgroups based on baseline scalp hair loss, including patients with AT and AU, with the highest treatment response observed in patients with 25% to 50% scalp hair loss.

## AUTHOR CONTRIBUTIONS

XL, EHL, RI‐A, RW, MS and AL contributed to the concept and design of the study. All authors contributed to data analysis and interpretation and critical revision of the publication, and all authors read and approved the final manuscript.

## FUNDING INFORMATION

This study was sponsored by Pfizer Inc.

## CONFLICT OF INTEREST STATEMENT

C Tziotzios: speaker for LEO Pharma; principal and chief investigator for Pfizer Inc; consultant for Pfizer Inc. R Sinclair: professional services from AbbVie, Aerotech, Amgen, Arena, Arcutis, AkseBio, AstraZeneca, Ascend, Bayer, Boehringer Ingelheim, BMS, Celgene, Coherus Biosciences, Cutanea, Connect, Dermira, Eli Lilly, Galderma, GSK, Janssen, LEO Pharma, MedImmune, Merck, MSD, Novartis, Oncobiologics, Pfizer Inc, Regeneron, Reistone, Roche, Sanofi, Samson Clinical, Sun Pharma and UCB. A Lesiak: speaker and advisory board member for Novartis, AbbVie, Sanofi, LEO Pharma, Eli Lilly, UCB and Pfizer Inc. S Mehlis: received clinical trial support from AbbVie, Akari, Amgen, AstraZeneca, BMS, ChemoCentryx, Dong‐A ST, Eli Lilly, Galderma, Incyte, Janssen, LEO Pharma, Menlo, Mayne Pharma, NFlection, Nimbus, Novartis, Pfizer Inc, Regeneron, Sanofi, UCB, Vanda and VYNE; speaker, advisor or consultant for AbbVie, Janssen, Novartis, Dermavant, LEO Pharma and UCB. M Kinoshita‐Ise: clinical trial investigator and speaker for Eli Lilly and Pfizer Inc; clinical trial investigator for BMS and AbbVie. A Tsianakas: clinical trial investigator and speaker for Pfizer Inc. X Luo, EH Law, R Ishowo‐Adejumo, R Wolk, M Sadrarhami and A Lejeune: employees of and hold stock or stock options in Pfizer Inc.

## ETHICAL APPROVAL

The protocol was reviewed and approved by the institutional review boards or ethics committees of the participating institutions. The study was conducted in accordance with the International Ethical Guidelines for Biomedical Research Involving Human Subjects (Council for International Organizations of Medical Sciences 2002), International Council for Harmonisation Guideline for Good Clinical Practice and the Declaration of Helsinki.

## ETHICS STATEMENT

All participants provided informed consent.

## Supporting information


Data S1.

## Data Availability

Upon request, and subject to review, Pfizer will provide the data that support the findings of this study. Subject to certain criteria, conditions and exceptions, Pfizer may also provide access to the related individual de‐identified participant data. See https://www.pfizer.com/science/clinical‐trials/trial‐data‐and‐results for more information.
