# Supplementary material for: Long‐term safety and efficacy of ritlecitinib in adults and adolescents with alopecia areata and at least 25% scalp hair loss: Results from the ALLEGRO‐LT phase 3, open‐label study
Source: J Eur Acad Dermatol Venereol. 2025 Jan 23;39(6):1152–62. doi: 10.1111/jdv.20526 (PMC12105460; doi:10.1111/jdv.20526)
Supplement: Supplementary file 1 — Data S1. [file JDV-39-1152-s001.docx]

**SUPPLEMENTAL MATERIALS**

**Table S1.** Inclusion and exclusion criteria.

| **Inclusion criteria** | **Exclusion criteria** |
| --- | --- |
| - Age ≥12 years - <18 years if permitted by the sponsor, local competent authority, and IRB/IEC - Agree to avoid prolonged sun exposure and not use tanning booths, sun lamps, or other UV light sources during the study - Meet reproductive criteria, including relevant contraceptive methods - Rollover patients from NCT03732807 must have completed ≥34 weeks of study intervention - *De novo* or rollover patients >30 days from the first study visit of study and the last dose in studies NCT02974868 or NCT03732807 had to meet the following AA criteria: - Clinical diagnosis of AA with no other etiology of hair loss; androgenetic alopecia coexistent with AA was allowed provided the below threshold for hair loss due to AA was met - Hair loss carefully reviewed to verify the required percentage of terminal scalp hair loss is due to AA - ≥25% hair loss of the scalp, including AT and AU - No evidence of terminal hair regrowth within 6 months at both screening and BL visits (*de novo* only) - Current episode of hair loss ≤10 years (*de novo* only) | - Other scalp disease or active systemic disease - Any psychiatric condition, including recent or active suicidal ideation or behavior that meets any of the listed protocol criteria - Auditory conditions considered acute, fluctuating, or progressive - Known immunodeficiency disorder, including positive serology for HIV at screening - Present/past malignancies, except for adequately treated or excised nonmetastatic basal cell or squamous cell cancer of the skin or cervical carcinoma in situ - Past/present lymphoproliferative disorder, lymphoma, or leukemia - History (single episode) of disseminated HZ or disseminated herpes simplex, or recurrent (≥1 episode) of localized, dermatomal HZ - Current/recent history of clinically significant severe, progressive, or uncontrolled renal, hepatic, hematologic, gastrointestinal, metabolic, endocrine, pulmonary, cardiovascular, psychiatric, immunological/rheumatological, or neurological disease - Age 12 to <18 years without a documented history of VZV vaccination or presence of VZV IgG Ab - History of systemic infection, active acute or chronic infection, or infection with HBV or HCV - History of systemic infection requiring hospitalization within 6 months; active acute or chronic infection requiring treatment with oral antibiotics, antivirals, antiparasitics, antiprotozoals, or antifungals within 4 weeks prior to Day 1; history of either untreated or inadequately treated latent or treated for active TB infection - Anticipated treatment with prohibited concomitant medication(s) during the course of the study. Participation in studies other than NCT02974868 or NCT03732807 involving investigational drug(s) within 8 weeks (12 weeks for JAK inhibitors other than ritlecitinib received in NCT02974868 or NCT03732807) or within 5 half-lives (if known), whichever is longer, prior to study entry and/or during study participation - Received other treatment regimens in the specified time frames (as listed above for NCT03732807) |

AA, alopecia areata; Ab, antibody; AT, alopecia totalis; AU, alopecia universalis; BL, baseline; HBV, hepatitis B virus; HCV, hepatitis C virus; HZ, herpes zoster; IEC, independent ethics committee; IgG, immunoglobulin G; IRB, institutional review board; TB, tuberculosis; VZV, varicella-zoster virus.

**Table S2**. Prohibited concomitant medications and treatments.

| **Medications and treatments that could affect AA** |
| --- |
| JAK inhibitors for use in any disease indication |
| Immunosuppressants (e.g., cyclosporine A, azathioprine, MTX, sulfasalazine, MMF, everolimus, ibrutinib) |
| Intralesional, oral, or injectable steroids |
| 5-ARIs (e.g., finasteride, dutasteride) unless used for androgenetic alopecia |
| Oral minoxidil |
| Spironolactone unless taken for androgenetic alopecia or a medical condition  other than AA (e.g., hypertension) |
| Other systemic treatments that could affect AA |
| Topical steroids (e.g., steroid cream, steroid ointment) on areas under assessment  (i.e., scalp, eyebrows, eyelashes, and fingernails) |
| Topical treatments (e.g., medicated shampoo, minoxidil) that could affect AA on  areas under assessment (i.e., scalp, eyebrows, eyelashes, and fingernails) unless  used for androgenetic alopecia |
| Phototherapy (e.g., UVB phototherapy, PUVA) |
| Contact immunotherapy (e.g., DPCP, SADBE, and DNCB) |
| Topical irritants (e.g., anthralin) and liquid nitrogen cryotherapy |
| Cosmetic treatments/applications |
| **Medications with potential drug-drug interactions or potential safety concerns** |
| Lymphocyte-depleting agents/therapies, including both non-B-cell–selective and B-cell–depleting agents (e.g., alefacept, alemtuzumab, rituximab) |
| Other biologics with immunomodulatory properties |
| Live (attenuated) vaccines |
| Moderate to potent CYP3A inducers |
| Specifically sensitive to moderately sensitive CYP3A substrates |
| Herbal medications with either unknown properties or pharmaceutical properties that impact AA |
| Investigational products (e.g., drugs or vaccines) |

5-ARI, 5α-reductase inhibitor; AA, alopecia areata; CYP3A, cytochrome p450, family 3, subfamily A; DNCB, 1-chloro-2,4-dinitrobenzene; DPCP, diphenylcyclopropenone; MMF, mycophenolate mofetil; MTX, methotrexate; PUVA, psoralen plus ultraviolet A; SADBE, squaric acid dibutylester; UVB, ultraviolet B.
